# Editorial: Digital health adoption: Looking beyond the role of technology

**DOI:** 10.3389/fdgth.2022.989003

**Published:** 2022-11-07

**Authors:** Yiannis Kyratsis, Harry Scarbrough, Amanda Begley, Jean-Louis Denis

**Affiliations:** ^1^ Department of Organization Sciences, Faculty of Social Sciences, Vrije Universiteit Amsterdam, Amsterdam, Netherlands; ^2^ Bayes Business School, City, University of London, London, United Kingdom; ^3^ Centre for Innovation, Transformation and Improvement, Guy’s & St Thomas’ NHS Foundation Trust, London, United Kingdom; ^4^ École de Santé Publique, Université de Montréal, Montreal, QC, Canada

**Keywords:** digital health, technology adoption, health innovation, organization & administration, implementation, health managament

**Editorial on the Research Topic** Digital health adoption: Looking beyond the role of technology By Kyratsis Y, Scarbrough H, Begley A and Denis J-L. (2022) Front. Digit. Health. 4: 989003. doi: 10.3389/fdgth.2022.989003

Accelerating the adoption of proven digital health technologies and advancing their embedding into routine care operations has the potential to revolutionize human health by boosting efficacy, driving costs down, and increasing access to and capacity for care delivery (1). It can shape individuals' daily lifestyle choices, and advance population health management, thus improving life expectancy and quality of life worldwide (2). Nonetheless, the healthcare sector has been struggling to accelerate digital adoption. In line with recent insights in the literature (3, 4), the papers in this Research Topic illustrate that much of this frustration relates to challenges that lie beyond managing the technologies' technical core. We first introduce the papers and then reflect on key themes and implications.

Reflecting on adoption Shaw and Donia propose a broader socio-technical approach to the ethics of digital health, which spans domains from software, devices and supply chains to inter-personal relationships, organizational and government policies. They emphasize issues of social justice, the need to address inequalities in digital access and advocate anticipatory forms of governance to minimize potential negative consequences. Greenhalgh et al. discuss a conceptual framework—PERCS—used to evaluate remote healthcare consultation services in the UK. The authors focus attention on digital maturity and digital inclusion, examining seven inter-related domains, spanning from the reason for consultation, to patients, care delivery, home and family and the wider system. They identify tensions and contradictions along these domains and elaborate on related practical ethical issues. Shaw et al. analyze the accelerated implementation of video consulting during the COVID-19 pandemic. Using comparative and interpretive policy analysis, the authors identify key variations across the four UK health systems in terms of enabling and limiting conditions at both policy and delivery levels. The authors also caution against inequalities in accessing video consulting services.

Nantume et al. explore the commercialization of a wearable vital signs monitor in low resource settings and argue for a holistic implementation perspective, from idea and product design to market. They highlight implementation being intertwined with development and evaluation, involving local stakeholders as co-creators. The authors also stress the role of social dynamics, such as trust in regulatory authorities, and public misperceptions about the technology. Rainey et al. in their survey study explore perceptions of AI by UK radiographers for successful application and integration into clinical practice. The authors highlight important aspects of the professional roles of clinicians, and the need for learning, capability-building and de-mystification of the opacity of AI-in use. Bouabida et al. evaluated two platforms for remote patient monitoring following hospital discharge in the context of COVID-19 in Canada. The authors highlight issues of social acceptability by diverse stakeholders during adoption, maintaining human contact and balancing concerns for confidentiality and data security. They underline the need for user participation in technology development and deployment, also bringing to the fore organizational, social and ethical aspects.

Bennet et al. describe the experience of ID-Liver implementation and use in northern England for integrated, pro-active management of patients at risk of developing chronic liver disease. From setting up to piloting and using ID-Liver, the authors argue for the need to mobilize a network of collaborators including commercial partners, healthcare organizations and professionals. Yan et al. in their commentary on digital therapeutics raise the broader question of cost and reimbursement. The authors identify a set of dilemmas for policy-makers, which are related to the specificities of digital therapeutics including the ability of patients to afford and use technological devices and the possibility of reimbursing these therapeutics. Cripps and Scarbrough present a perspective on a sustainable approach to digital applications in the UK's NHS. The authors argue to shift the focus from the technology itself to considering the motivations of users and constraints within specific contexts. They advocate for a wider approach to change that incorporates clinical and behavioral insights, process engineering and knowledge management.

**Figure 1:**
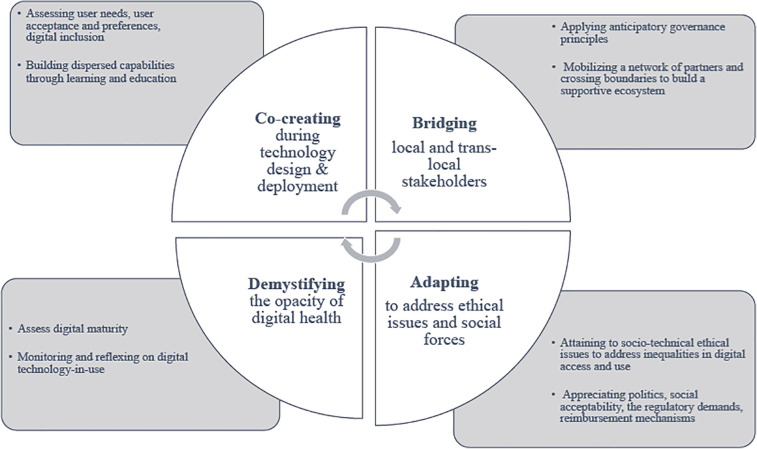
Themes on digital health adoption.

The papers' contribution can be grouped into four themes, which highlight key non-technology related aspects of digital health adoption (Figure 1): (a) *Co-creating* through digital inclusion and user engagement. (b) *Bridging local and trans-local stakeholders* including partners from the wider economy and the private sector. (c) *Adapting to ethical issues and social forces,* beyond technical and clinical aspects, such as public (mis-)perceptions, professional and organizational dynamics, regulatory elements, as well as issues of cost and reimbursement. (d) *Demystifying the opacity* (clinical, operational) of digital health applications and assessing digital maturity in practice.

Reflecting on implications for policy and practice, the papers in this Research Topic highlight *five key levers* that can help drive more effective digital health technology adoption.

1. *Understanding and responding to the needs and preferences of diverse individuals and communities* is critical (e.g., Cripps and Scarbrough). A number of authors (e.g., Shaw and Donia; Greenhalgh et al.) highlight inequalities in digital health. While inequality is often considered at the point of care in terms of the ability of patients and clinicians to use technology, inequalities also arise when more marginalized groups are unable to voice their concerns and preferences upstream, and to influence the development and evaluation of digital innovations. Aptly, the question of *co-creation* underpins many papers in the collection.
2. *Early and active stakeholder engagement in both design and technology use* (e.g., Nantume et al.). This highlights the need to partner with and incentivize innovators (including the private sector) to bring in their technical expertise (e.g., Bennet et al.), as well as effective collaboration with patients, healthcare providers and commissioners.
3. *Building the capability and confidence of all actors to acknowledge and raise quality, privacy, security and safety concerns* relating to digital health care (e.g., Shaw et al. Bouabida et al.). Reskilling, learning and modifying professional roles play a vital role in adoption as the Rainey et al. paper illustrates.
4. *Adopting a holistic, rather than a piecemeal approach to build a supportive ecosystem*. This suggests the need for a long-term strategy, appreciating politics, the regulatory groundwork, reimbursement mechanisms, cost (Yan et al.).
5. *Considering seriously the wider ethical implications* of digital health (e.g., Shaw and Donia) to establish and maintain trust, transparency and accountability.
